# The enzyme pseudooxynicotine amine oxidase from *Pseudomonas putida* S16 is not an oxidase, but a dehydrogenase

**DOI:** 10.1016/j.jbc.2022.102251

**Published:** 2022-07-11

**Authors:** Vishakha Choudhary, Kevin Wu, Zhiyao Zhang, Mark Dulchavsky, Todd Barkman, James C.A. Bardwell, Frederick Stull

**Affiliations:** 1 Department of Chemistry, Western Michigan University, Kalamazoo, Michigan, USA; 2 Howard Hughes Medical Institute, University of Michigan, Ann Arbor, Michigan, USA; 3 Department of Biophysics, University of Michigan, Ann Arbor, Michigan, USA; 4 Cellular and Molecular Biology Program, University of Michigan, Ann Arbor, Michigan, USA; 5 Department of Biological Sciences, Western Michigan University, Kalamazoo, Michigan, USA; 6 Department of Molecular, Cellular and Developmental Biology, University of Michigan, Ann Arbor, Michigan, USA

**Keywords:** flavoenzyme, flavoprotein, flavin, cytochrome c, pseudooxynicotine, nicotine, dehydrogenase, oxidase, enzyme kinetics, ETF, electron transferring flavoprotein, FAD, flavin adenine dinucleotide, FADH2, reduced flavin hydroquinone, LHNO, L-6-hydroxynicotine oxidase, NicA2, nicotine oxidoreductase, Pnao, pseudooxynicotine amine oxidase, Pon, pseudooxynicotine

## Abstract

The soil-dwelling bacterium *Pseudomonas putida* S16 can survive on nicotine as its sole carbon and nitrogen source. The enzymes nicotine oxidoreductase (NicA2) and pseudooxynicotine amine oxidase (Pnao), both members of the flavin-containing amine oxidase family, catalyze the first two steps in the nicotine catabolism pathway. Our laboratory has previously shown that, contrary to other members of its enzyme family, NicA2 is actually a dehydrogenase that uses a cytochrome c protein (CycN) as its electron acceptor. The natural electron acceptor for Pnao is unknown; however, within the *P. putida* S16 genome, *pnao* forms an operon with *cycN* and *nicA2*, leading us to hypothesize that Pnao may also be a dehydrogenase that uses CycN as its electron acceptor. Here we characterized the kinetic properties of Pnao and show that Pnao is poorly oxidized by O_2_, but can be rapidly oxidized by CycN, indicating that Pnao indeed acts as a dehydrogenase that uses CycN as its oxidant. Comparing steady-state kinetics with transient kinetic experiments revealed that product release primarily limits turnover by Pnao. We also resolved the crystal structure of Pnao at 2.60 Å, which shows that Pnao has a similar structural fold as NicA2. Furthermore, rigid-body docking of the structure of CycN with Pnao and NicA2 identified a potential conserved binding site for CycN on these two enzymes. Taken together, our results demonstrate that although Pnao and NicA2 show a high degree of similarity to flavin containing amine oxidases that use dioxygen directly, both enzymes are actually dehydrogenases.

*Pseudomonas putida* S16, a gram-negative bacteria isolated from the soil of a tobacco field in Shandong, China, has the remarkable property of being able to grow on nicotine as a sole carbon and nitrogen source (1). The biochemical pathway through which *P. putida* S16 degrades nicotine is known to involve enzyme catalyzed steps that catabolize nicotine, eventually resulting in fumarate and ammonia, which are used for primary metabolism (1). The first two steps in the pathway are catalyzed by the enzymes nicotine oxidoreductase (NicA2) and pseudooxynicotine amine oxidase (Pnao), which convert nicotine into pseudooxynicotine (Pon) and Pon into 3-succinoylsemialdehyde-pyridine, respectively (Fig. 1) (2, 3). Both NicA2 and Pnao are members of the widespread flavin-dependent amine oxidase enzyme family. Enzymes of this family use a flavin adenine dinucleotide (FAD) prosthetic group to accept a hydride from their amine containing substrate, forming an oxidized product and reduced flavin hydroquinone (FADH_2_) in the reductive half reaction (4). For subsequent rounds of catalysis to occur, the electrons on the enzyme bound FADH_2_ must be passed to an electron acceptor to regenerate oxidized FAD in the oxidative half reaction. Members of the flavin-dependent amine oxidase family are generally assumed to use molecular oxygen (O_2_) as their electron acceptor, producing H_2_O_2_ as a second product (4, 5). However, our lab recently demonstrated that NicA2 is instead a dehydrogenase that uses a cytochrome c protein (called CycN) as a biological oxidant (6). This makes NicA2 a notable exception to the oxidase paradigm of the flavin-dependent amine oxidase family. The electrons that are passed from NicA2 to CycN are then presumably transferred to a membrane-bound cytochrome c oxidase for additional energy production.

**Figure 1:**
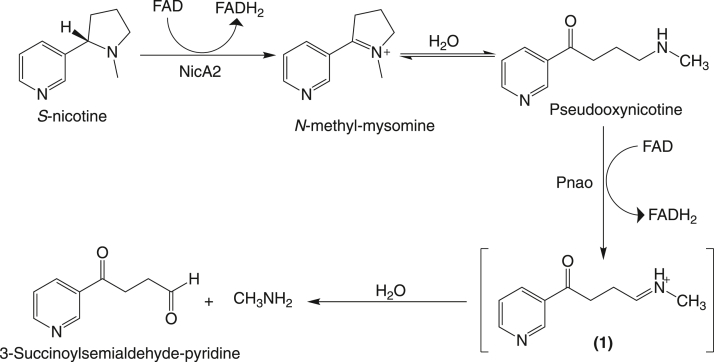
**Pathway for the first two steps of nicotine catabolism catalyzed by NicA2 and Pnao in *P.putida*S16**.

The *nicA2* gene forms an operon with *cycN* and *pnao* in the genome of *P. putida* S16, indicating that these three genes are likely coregulated as part of a functional unit (6). All three proteins are also predicted to contain N-terminal signal sequences for export to the periplasm, localizing them in the same subcellular compartment. These two points led us to wonder if Pnao, which only shares 39% sequence identity to NicA2, is also a dehydrogenase that uses CycN as its biological electron acceptor in *P. putida* S16. In this study, we demonstrate that Pnao-containing FADH_2_ is poorly oxidized by O_2_, yet can be rapidly oxidized by CycN, indicating that Pnao is indeed a dehydrogenase. The ability to transfer electrons to CycN is not a general attribute of flavin-dependent amine oxidases, as the related enzyme L-6-hydroxynicotine oxidase (LHNO) from *Arthrobacter nicotinovorans* is poorly oxidized by CycN. Comparing parameters from steady-state kinetics and transient kinetics reveals that release of product from Pnao after Pon oxidation is primarily rate limiting during turnover at saturating CycN concentrations. The structure of Pnao, which we succeeded in resolving to 2.60 Å, shows a similar overall architecture as NicA2, with the isoalloxazine of the FAD buried near the hydrophobic active site cavity that accommodates Pon, and rigid-body docking reveals a potential conserved binding site for CycN on the surface of Pnao and NicA2. Overall, our results demonstrate that multiple members of the flavin-dependent amine oxidase family—NicA2 and Pnao—are actually dehydrogenases that use CycN as an oxidant.

## Results

### Pnao has an air stable flavin semiquinone

**Figure 2:**
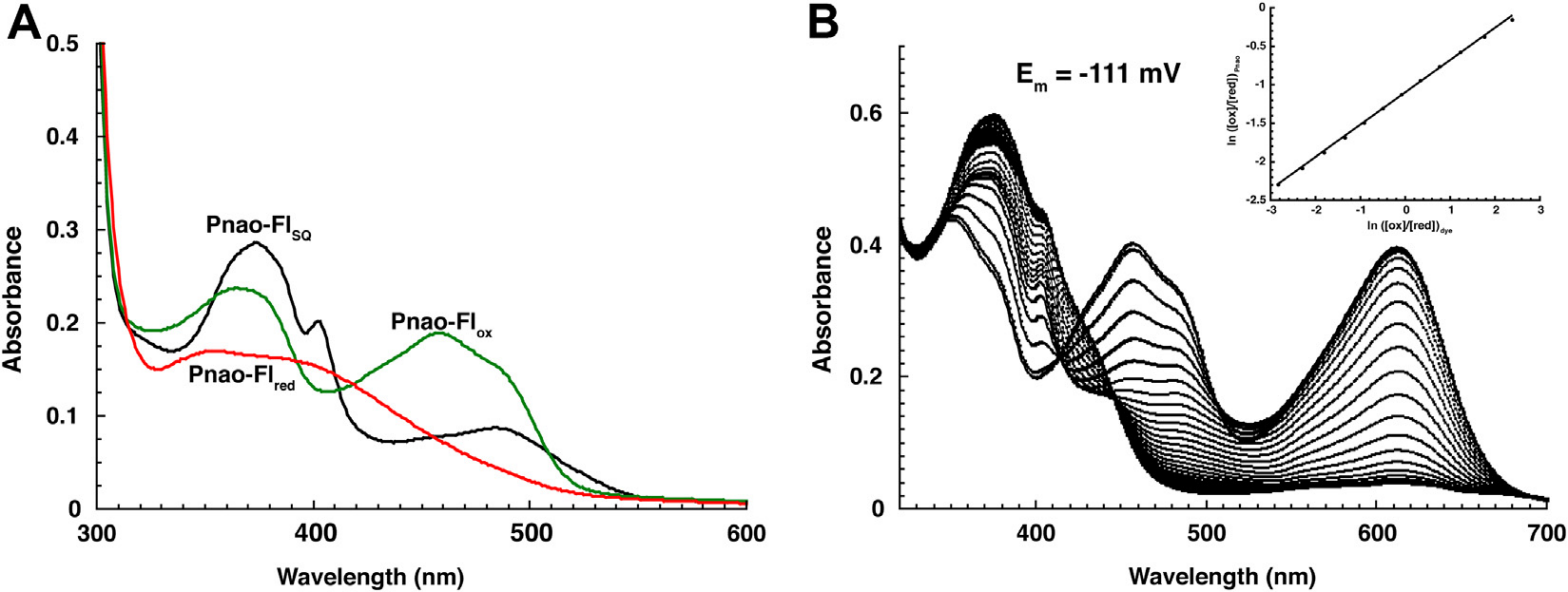
**Absorbance and redox properties of Pnao.***A*, UV/visible absorbance spectra of Pnao containing oxidized FAD (Pnao-Fl_ox_), Pnao containing anionic FAD semiquinone (Pnao-Fl_SQ_) and Pnao containing FADH_2_ (Pnao-Fl_red_). Pnao-Fl_SQ_ and Pnao-Fl_red_ were produced by anaerobic reduction of Pnao-Fl_ox_ with dithionite and pseudooxynicotine, respectively. *B*, absorbance data used to calculate reduction potential for Pnao-Fl_ox_/Pnao-Fl_SQ_ under anaerobic conditions at pH 7.5, 25 °C. Spectra for reduction of enzyme and the indicator dye indigo disulfonate were collected every 15 to 30 min. The indicator dye provides the large absorbance at 600 nm. Inset shows the Nernst plot yielding a reduction potential of E_m_= −111 mV. FAD, flavin adenine dinucleotide; Pnao, pseudooxynicotine amine oxidase.

During routine purification of Pnao from *Escherichia coli*, we observed that the enzyme obtained after the first Ni^2+^ affinity step lacked the bright yellow color that is generally observed with flavin-containing proteins. The UV/Visible absorbance spectrum of the enzyme at this stage revealed spectral features diagnostic of a one-electron reduced anionic flavin semiquinone (Fig. 2*A*) (7). Pnao containing this flavin semiquinone (Pnao-Fl_SQ_) was stable under aerobic conditions for at least an hour. Stabilization of the flavin semiquinone indicates that the reduction potential for the oxidized/semiquinone couple is higher than the potential for the semiquinone/hydroquinone couple in the FAD bound to Pnao and that there is a wide separation between the reduction potentials for these two couples. Exposure to air overnight resulted in conversion to the absorbance spectrum of oxidized flavin, and the fully oxidized form of the enzyme was used for all subsequent experiments. The component of *E. coli* lysates that reduces Pnao is unknown, but the fact that Pnao’s flavin semiquinone is remarkably air stable provided the first indication that Pnao reacts poorly with O_2_.

Pnao’s flavin semiquinone was also remarkably resistant to reduction by nonphysiological reductants. Anaerobic treatment of Pnao containing oxidized FAD (Pnao-Fl_ox_) with dithionite reduced the enzyme to Pnao-Fl_SQ_ but was unable to reduce the flavin further to the hydroquinone (Pnao-Fl_red_) even after incubating with excess dithionite for several hours. Likewise, anaerobic reduction of the enzyme using the xanthine/xanthine oxidase system was only able to reduce the flavin to Pnao-Fl_SQ_ with no further reduction to Pnao-Fl_red_. We measured the reduction potential of the Pnao-Fl_ox_/Pnao-Fl_SQ_ couple using the xanthine/xanthine oxidase method developed by V. Massey (8) using indigo disulfonate as the indicator dye, which has a reduction potential of -139 mV at pH 7.5 and 25 °C. The linearized Nernst plot produced a reduction potential of -111 mV for the Pnao-Fl_ox_/Pnao-Fl_SQ_ couple (Fig. 2*B*). We were unable to determine the reduction potential for the Pnao-Fl_SQ_/Pnao-Fl_red_ or Pnao-Fl_ox_/Pnao-Fl_red_ couples due to the inability to reduce the enzyme past the semiquinone using nonphysiological reductants.

### Pnao reduction by Pon

In the reductive half-reaction for Pnao, enzyme containing oxidized FAD is reduced by Pon to produce flavin hydroquinone and the imine-containing intermediate **1** shown in Figure 1. The imine-containing intermediate is unstable and undergoes spontaneous hydrolysis to form 3-succinoylsemialdehyde-pyridine and methylamine as the final products in the reaction of Pon with Pnao. We studied the kinetics of the reductive half reaction for Pnao in anaerobic stopped-flow experiments by mixing Pnao-Fl_ox_ with Pon in the absence of O_2_ and used the instrument’s multiwavelength charge coupled device detector to monitor the progress of the reaction.

**Figure 3:**
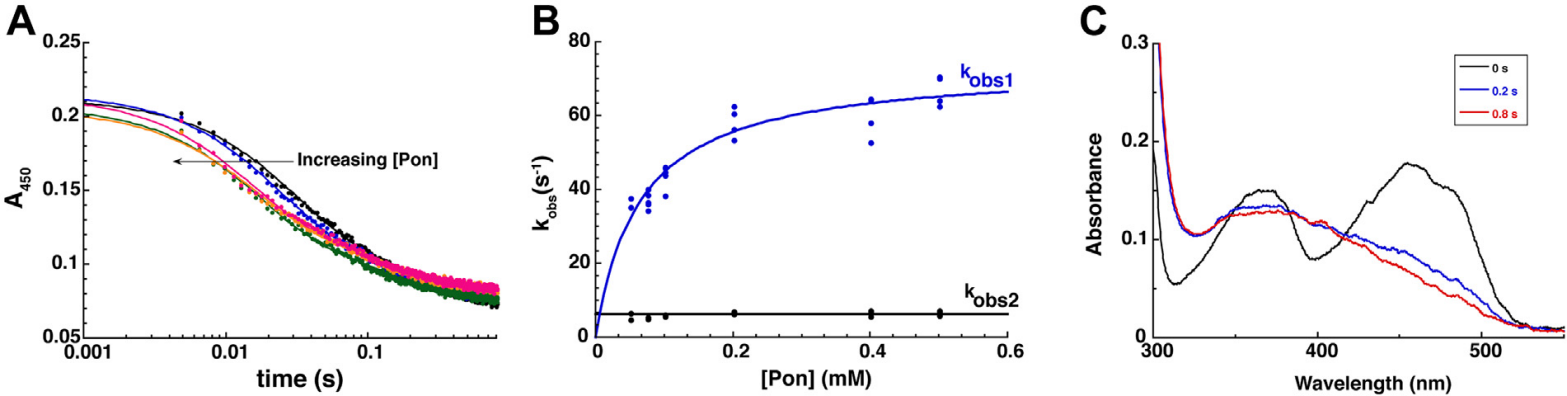
**Kinetics of Pnao reduction by pseudooxynicotine.***A*, absorbance trace overlay at 450 nm for the reduction of Pnao-Fl_ox_ by various concentrations of pseudooxynicotine. Note the logarithmic time scale. *B*, k_obs_ values for the first and second phase plotted against the concentration of pseudooxynicotine. The first phase, which showed a hyperbolic dependence, was fitted to Equation 5 to yield k_red_ of 74 ± 3 s^-1^ and a K_d_ of 64 ± 8 μM for pseudooxynicotine. The k_obs_ value for the second phase was invariant with pseudooxynicotine concentration at 6.3 ± 0.2 s^−1^. *C*, spectral changes observed at different times during the reduction of Pnao-Fl_ox_ by pseudooxynicotine. Pnao, pseudooxynicotine amine oxidase.

The reaction completed in 0.8 s and reaction traces at 450 nm fit best to two exponentials (Fig. 3, *A* and *B*), with the first phase contributing ∼65% of the total signal change at this wavelength. The absorbance spectrum at 0.2 s, the approximate transition point between the two kinetic events, indicated that the first observable kinetic event corresponds to reduction of Pnao’s flavin by Pon (Fig. 3*C*). We attribute the subsequent, second phase to release of the imine-containing product (**1** in Fig. 1) that results from oxidizing Pon. The observed rate constant (k_obs_) for the first phase varied hyperbolically with the Pon concentration, consistent with this step reporting on hydride transfer from Pon to Pnao’s flavin after the complex has formed. From the hyperbolic fit of the k_obs_ data for phase one, we determined that the rate constant for flavin reduction (k_red_) was 74 ± 3 s^-1^, with an apparent K_d_ of 64 ± 8 μM for binding Pon. The k_obs_ for the second phase was invariant with Pon concentration, giving a rate constant for dissociation of the imine-containing product of 6.3 ± 0.2 s^-1^.

### Pnao reacts slowly with O_2_

**Figure 4:**
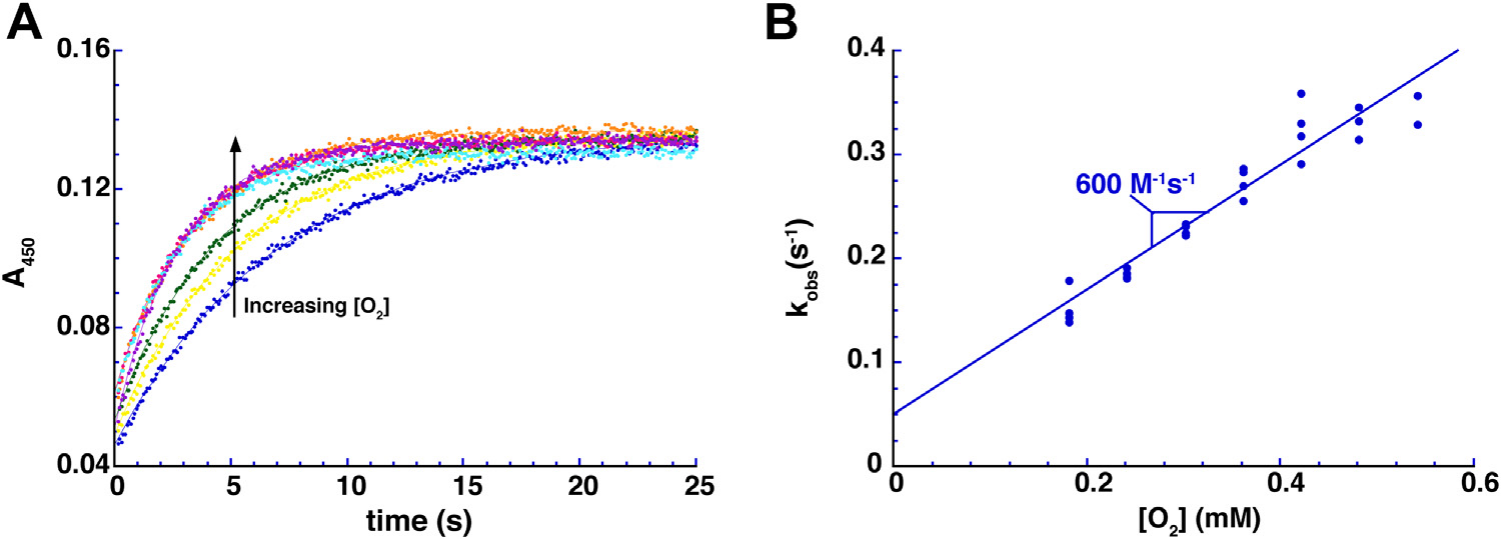
**Oxidation of reduced Pnao by O**_**2**_**.***A*, absorbance traces at 450 nm for the oxidation of Pnao-Fl_red_ by various concentrations of O_2_. *B*, plot of k_obs_ against the O_2_ concentration. Pnao, pseudooxynicotine amine oxidase.

The reaction between Pnao-Fl_red_ and O_2_ was monitored in stopped-flow experiments. As previously mentioned, attempts at preparing Pnao-Fl_red_ with the commonly used reductant, dithionite, were unsuccessful as dithionite was unable to reduce Pnao-Fl_SQ_ to Pnao-Fl_red_ even after incubating for several hours. Instead, we prepared Pnao-Fl_red_ by anaerobically titrating Pnao-Fl_ox_ with one equivalent of Pon in a tonometer. Pnao-Fl_red_ was then mixed with buffer containing various concentrations of O_2_, and the reoxidation reaction was monitored using a multiwavelength absorbance detector. In contrast to the reductive half reaction, oxidation of Pnao-Fl_red_ by O_2_ was dramatically slower, taking ∼20 s (Fig. 4). Pnao-Fl_red_ directly reoxidized into Pnao-Fl_ox_ without any detectable intermediates. Kinetic traces at 450 nm were fit to a single exponential and k_obs_ for the reaction increased linearly with increasing O_2_ concentrations. Linear fitting of the k_obs_ plot produced a bimolecular rate constant for flavin oxidation (k_ox_^O2^) of 600 ± 40 M^-1^s^-1^. While this value is about 20-fold greater than the k_ox_^O2^ previously determined for NicA2 (28 M^-1^s^-1^), it is still considerably lower than that of *bona fide* flavin-dependent amine oxidases, which usually have k_ox_^O2^ values on the order of 10^4^−10^6^ M^−1^s−^1^ (9).

### Pnao is rapidly oxidized by CycN

**Figure 5:**
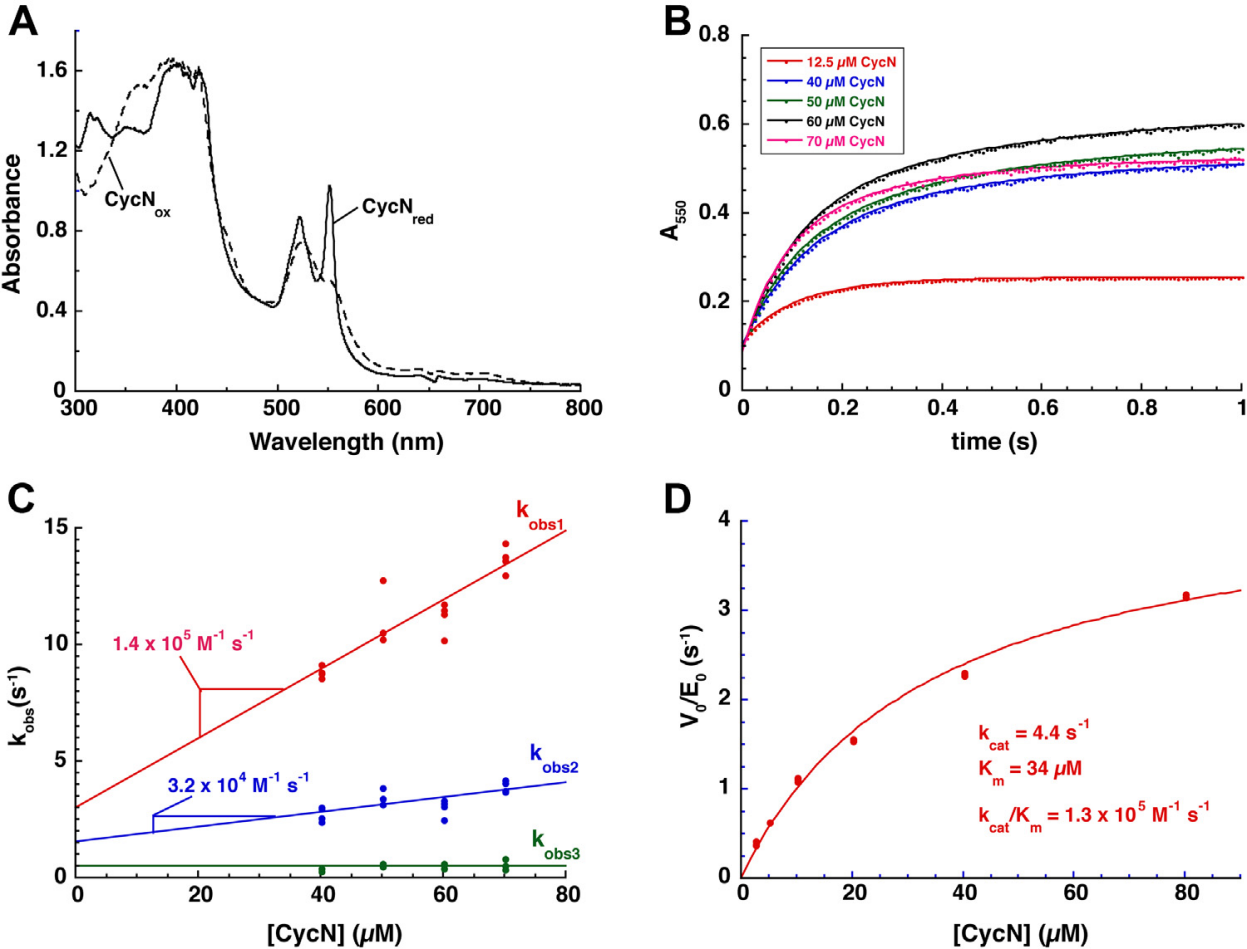
**Reaction of reduced Pnao with oxidized CycN.***A*, spectral overlay comparing absorbance changes between CycN_ox_ (*dashed line*) and CycN_red_ (*solid line*) during reaction with reduced Pnao. Note that absorbance values above 1.5 are inaccurate due to saturation of the CCD detector. *B*, absorbance traces at 550 nm from stopped-flow experiments where Pnao-Fl_red_ was mixed with various concentrations of oxidized CycN. To facilitate comparison, the traces were manually adjusted such that they all begin at the same absorbance value. *C*, the k_obs_ values plotted against CycN concentrations. The k_obs_ values for first and second phases demonstrate a linear dependence on [CycN]. k_obs3_ is invariable at 0.5 s^−1^ with changing CycN concentrations. *D*, steady-state velocity data for the Pnao-catalyzed reduction of CycN_ox_ using a saturating concentration of pseudooxynicotine. Pnao, pseudooxynicotine amine oxidase.

CycN is the natural electron acceptor for NicA2, and the *cycN* gene forms an operon with *nicA2* and *pnao*. We therefore investigated if CycN is a good electron acceptor for Pnao through anaerobic stopped-flow experiments. Pnao-Fl_red_ (prepared by titrating with 1 equiv. of Pon) was anaerobically mixed with various concentrations of CycN, and the reaction monitored using a multiwavelength detector. The reaction was rapid, having completed in ∼1 s, and the spectral changes were consistent with the reduction of CycN’s ferric heme (CycN_ox_) to the ferrous state (CycN_red_) (Fig. 5*A*). Reaction traces at 550 nm, which report on reduction of CycN_ox_ to CycN_red_, fit best to three exponentials where the first two phases have similar amplitudes and, together, comprise ∼95% of the total signal change at this wavelength (Fig. 5*B*). This behavior is consistent with two stepwise one electron transfers from Pnao-Fl_red_ to two different CycN_ox_ molecules, with an obligate Pnao-Fl_SQ_ intermediate between the two one electron transfers. The origin of the small, third phase is unclear but may be due to a small population of damaged enzyme or an instrumentation artifact. Plots of k_obs_ against the CycN concentration showed that k_obs_ for the two major phases varied linearly with the CycN concentration, indicative of a bimolecular reaction for both kinetic events (Fig. 5*C*). The fact that the k_obs_ plots indicate a bimolecular reaction for both events indicates that binding of CycN_ox_ to Pnao-Fl_red_ and Pnao-Fl_SQ_ limits the rate of electron transfer in the first and second phase, respectively; electron transfer from Pnao’s flavin to CycN’s heme after the complex has formed must be much faster than binding. Effectively, CycN reacts with Pnao in a bimolecular reaction without forming a Michaelis complex, similar to how most flavin-dependent oxidases and monooxygenases react with O_2_ (9). Notably, similar behavior was observed previously in stopped-flow experiments for the reaction of reduced NicA2 with CycN_ox_ (6). Linear fitting of the k_obs_ plots produced bimolecular rate constants of 1.4 ± 0.2 × 10^5^ M^−1^ s^−1^ and 3.2 ± 0.9 × 10^4^ M^−1^ s^−1^ for the first and second phase, respectively. These values are 230-fold and 53-fold larger than k_ox_^O2^, indicating that CycN is a much better electron acceptor for Pnao than O_2_, in agreement with Pnao being a dehydrogenase.

### CycN is critical for growth of *P. putida* S16 on Pon

**Figure 6:**
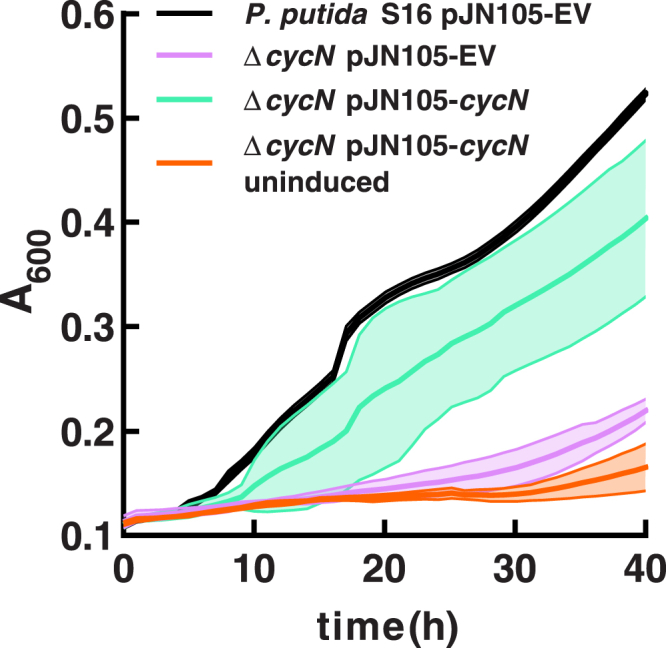
**CycN is required by *P. putida* S16 for robust growth on pseudooxynicotine.** Growth of the *P. putida* S16 strains was monitored by absorbance at 600 nm in a plate reader growth assay with pseudooxynicotine as a carbon source. Δ*cycN* in the legend refers to Δ*cycN P. putida* S16. EV denotes empty vector. Each curve is plotted as the mean of five biological replicates with error bands representing the standard deviation.

To further support that CycN is the *in vivo* electron acceptor for Pnao, we assessed the ability of *P. putida* S16 strains to grow using Pon as a carbon source. We previously observed that when grown using nicotine as a carbon source, *P. putida* S16 Δ*cycN* demonstrates a severe growth defect, consistent with the fact that NicA2 requires CycN to achieve rapid catalysis of nicotine (6). Without CycN present to facilitate reoxidation for NicA2, it is unable to degrade a sufficient amount of nicotine to enable robust growth. Similarly, when grown using Pon as a carbon source, *P. putida S16* Δ*cycN* has a comparable phenotype: the deletion strain grows slower than the wildtype, and this phenotype can be complemented by expressing cycN from a plasmid (Fig. 6). That the Δ*cycN* strain grows more slowly on Pon and is complementable by plasmid-borne expression of CycN, strongly supports that CycN serves as an *in vivo* electron acceptor for Pnao.

### Product release limits turnover by pnao

Steady-state kinetic parameters were determined for the Pnao catalyzed reduction of CycN_ox_ by a saturating concentration of Pon (1 mM) under anaerobic conditions. The steady-state data produced a K_M_ of 34 μM for CycN and a k_cat_ of 4.4 s^−1^ (Fig. 5*D*). This k_cat_ value is slightly lower than, but comparable to, the rate constant for product release determined in the reductive half reaction, suggesting that release of the imine-containing product is primarily rate-limiting during turnover with saturating concentrations of CycN. Hu *et al* previously determined a k_cat_ value of 0.8 s^−1^ for Pon oxidation by Pnao using ambient O_2_ as the oxidant (2); however, their experiments were performed at 30 °C and the O_2_-dependent k_cat_ value would likely be lower at 4 °C where our experiments were performed.

### Pnao is specific for CycN

**Figure 7:**
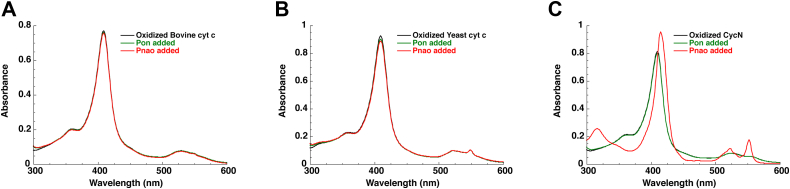
**Lack of reduction of bovine and yeast cytochrome *c* by Pnao.** (*A*), (*B*), and (*C*) bovine cytochrome *C*, yeast cytochrome c, and CycN, respectively, mixed stepwise with pseudooxynicotine and Pnao while monitoring the redox status of the cytochrome by UV/Visible absorbance. Pnao, pseudooxynicotine amine oxidase.

We wondered if Pnao was specific for CyN or if it would be able to reduce other cytochrome *c* proteins. Both bovine and yeast cytochrome *c*, which have 43% and 38% sequence identity to CycN, respectively, and are commercially available, were tested for their ability to accept electrons from Pnao. Neither bovine or yeast cytochrome c were reduced upon addition of Pnao and Pon, even after prolonged incubation (Fig. 7, *A* and *B*). In contrast, the stepwise addition of Pon and Pnao to CycN resulted in the reduction of CycN as expected (Fig. 7*C*). We previously observed that NicA2 and nicotine were unable to reduce bovine cytochrome c and noted that bovine cytochrome c has a much more positively charged surface than CycN on the heme-containing side of the protein (6). Yeast cytochrome c also has a similarly positively charged surface, and this difference in surface charge between CycN and bovine and yeast cytochrome c may, in part, be responsible for Pnao and NicA2’s specificity for CycN as a cytochrome c-based electron acceptor.

### CycN does not generally accept electrons from other flavoprotein amine oxidases

**Figure 8:**
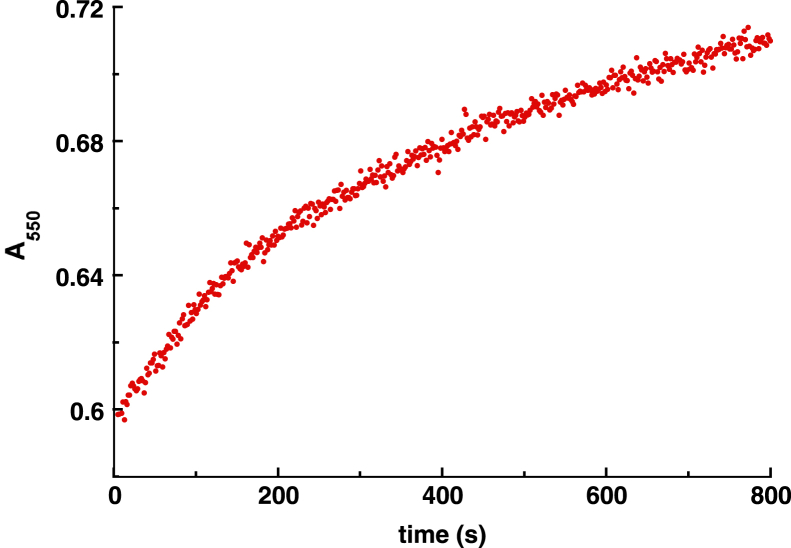
**Reaction of reduced LHNO with oxidized CycN.** An absorbance trace at 550 nm is shown for the anaerobic reaction of 15 μM reduced LHNO with 70 μM oxidized CycN. LHNO, L-6-hydroxynicotine oxidase.

The data presented here and in our prior report (6) indicate that CycN is a good recipient of electrons from NicA2 and Pnao, in agreement with both enzymes being dehydrogenases that use CycN as their oxidant. We wondered if CycN can rapidly oxidize not just these two proteins but also other members of the flavin-containing amine oxidase enzyme family. To investigate this possibility, we evaluated the enzyme LHNO from *A. nicotinovorans*, which is a closely related member of the flavin-containing amine oxidase family with potent oxidase activity (10, 11) for the ability to use CycN as an oxidant. To that end, anaerobic stopped-flow experiments were performed by mixing LHNO containing FADH_2_ (reduced using one equiv. of dithionite) with CycN_ox_, and the reaction was monitored over time. Notably, LHNO’s oxidized flavin reduced directly into the hydroquinone without any detectable semiquinone states during the titration with dithionite. Unlike NicA2 and Pnao, which are oxidized by CycN_ox_ in 1 s, reduced LHNO took >800 s to fully react with CycN_ox_ (Fig. 8), indicating that CycN is a poor recipient of electrons from LHNO. Therefore, the ability to rapidly transfer electrons to CycN appears not to be a general feature of all enzymes in the flavin-containing amine oxidase enzyme family.

### Structure of pnao

**Figure 9:**
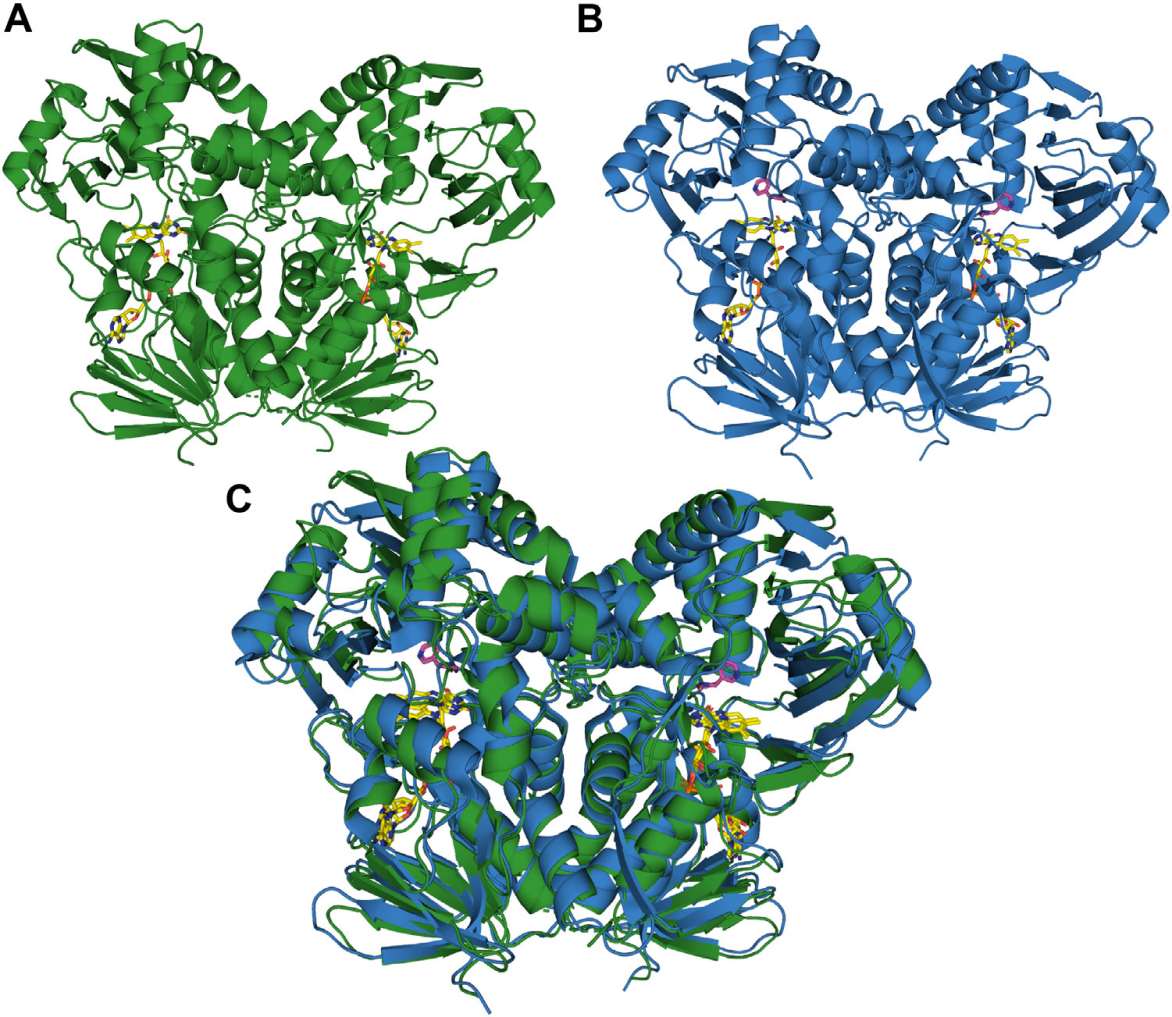
**Comparison of the overall structure of Pnao with NicA2.***A*, ribbon structure of Pnao. *B*, ribbon structure of NicA2 (PDB 6C71). *C*, overlay of the ribbon structures of Pnao and NicA2, colored as *green* and *blue*, respectively. In all structures, the FAD cofactor is shown in *yellow*. FAD, flavin adenine dinucleotide; NicA2, nicotine oxidoreductase; Pnao, pseudooxynicotine amine oxidase.

**Figure 10:**
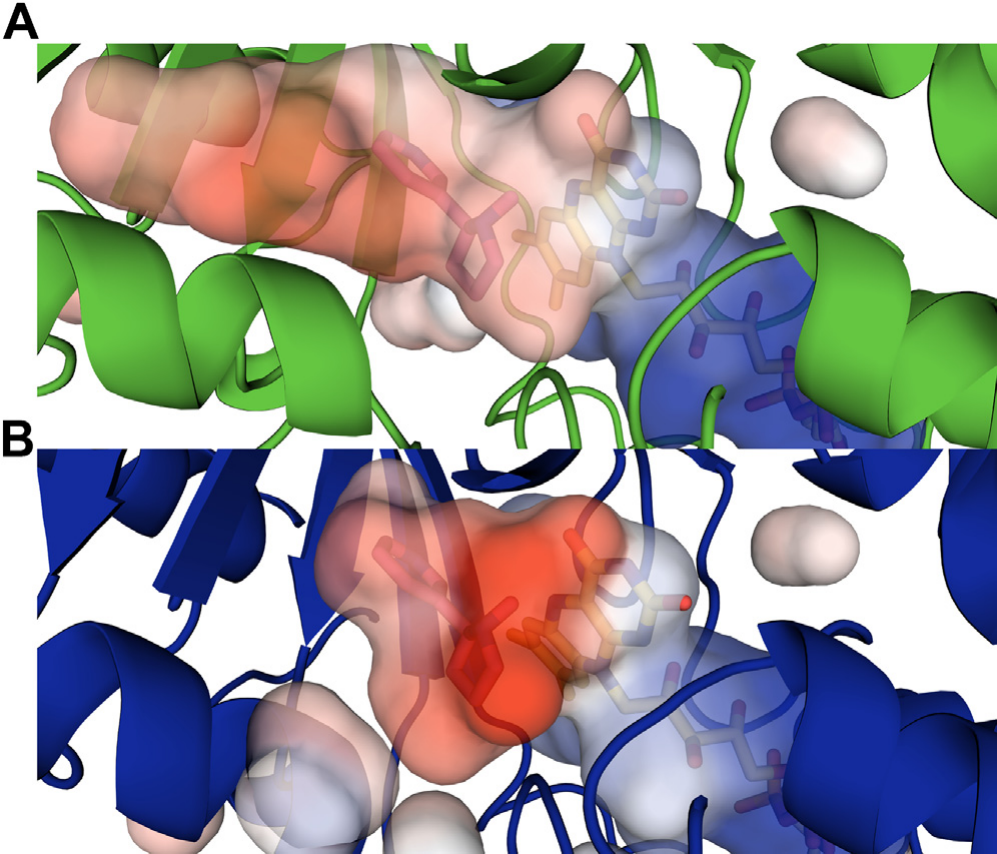
**Substrate binding pocket comparison between Pnao and NicA2.***A*, electrostatic potential map of the active site in Pnao. Nicotine has been placed for reference based on an alignment with the NicA2 structure. *B*, electrostatic potential map of the active site of NicA2 (PDB 6C71). In both structures, *(S)*-nicotine and FAD are shown as *magenta* and *yellow* stick models, respectively. FAD, flavin adenine dinucleotide; NicA2, nicotine oxidoreductase; Pnao, pseudooxynicotine amine oxidase.

The crystal structure of Pnao was solved at 2.60 Å resolution (Table S1). The structure of the Pnao monomer has a similar overall architecture as NicA2 (39% identity, RMSD of 1.5 Å), firmly placing Pnao in the flavoprotein amine oxidase enzyme family (Fig. 9). The asymmetric unit indicated that Pnao exists as a homodimer with a similar dimer interface as NicA2. Overlaying the Pnao structure with the nicotine-bound structure of NicA2 reveals that the substrate binding pocket in Pnao is considerably deeper than that in NicA2 due to a replacement of Trp364 in NicA2 with Ser372 in Pnao (Fig. 10). Trp364 forms the backstop of NicA2’s substrate binding site and forms hydrophobic interactions with the pyridine ring of nicotine; replacing it with Ser372 in Pnao allows for a more extended substrate binding cavity that is presumably better able to accommodate the more extended Pon substrate relative to nicotine (see Fig. 1).

**Figure 11:**
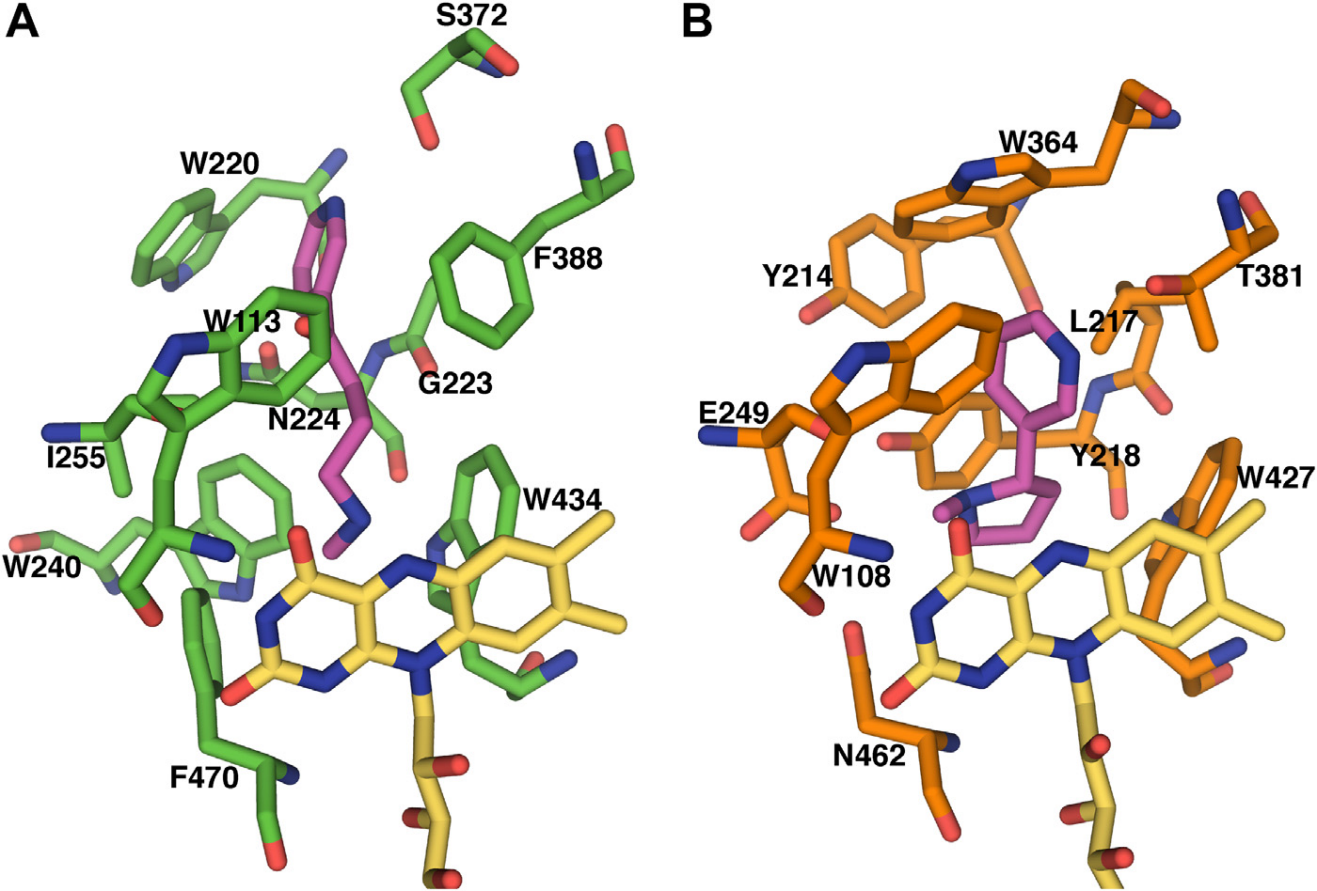
**Comparison of active site residues between Pnao and NicA2.***A*, docking model of pseudooxynicotine, shown in *magenta*, in the active site of Pnao. Active site residues are shown as *green sticks* and the FAD cofactor is shown in *yellow*. *B*, active site of NicA2 (PDB 6C71) with *(S)*-Nicotine shown in magenta. Active site residues are shown as *orange sticks*, and the FAD cofactor is shown in *yellow*. Note that the residue numbering for both enzymes is based on that of the full-length proteins, including the signal peptides. FAD, flavin adenine dinucleotide; NicA2, nicotine oxidoreductase; Pnao, pseudooxynicotine amine oxidase.

Another notable feature of the Pnao active site compared with NicA2 is the identity of the aromatic cage residues that surround the substrate. These aromatic cage residues flank the two ends of the isoalloxazine and have been shown to be important in proper positioning of the amine-containing substrate over N5 of the isoalloxazine in flavoprotein amine oxidases (12). In NicA2, the two residues that form the aromatic cage are Trp427 and Asn462, and this Asn residue is unusual and unique among this enzyme family. In Pnao, the aromatic cage residues are Trp434 and Phe470, which are more typical residues that compose the aromatic cage in this enzyme family. To facilitate comparison of the active sites of NicA2 and Pnao, we docked Pon into the active site of Pnao using AutoDock Vina (Fig. 11) (13). The two lowest energy docked structures (both –7.1 kcal/mol) had the pyridine ring of Pon oriented toward the flavin and were discarded; these are unlikely to be catalytically relevant because the methylamino group must be near FAD to be oxidized. The third lowest energy docked structure (–6.7 kcal/mol) had the methylamino end oriented toward the flavin with the carbon that transfers a hydride positioned 4.0 Å away from N5 of the isoalloxazine. This docking pose is shown in Figure 11*A*. We should note that this docking model is an approximation and that the actual position of Pon and the active site residues may be different than in the docking model. Several of the aromatic residues in the substrate binding site of NicA2 (Trp108, Tyr214, and Trp434) are the same or similar in Pnao (Trp113, Trp220, and Trp434) and may promote the hydrophobicity of the “aromatic cage” commonly found in flavoprotein amine oxidases. As previously mentioned, Trp364, which forms the end of the nicotine binding pocket in NicA2 is replaced with Ser372 in Pnao. Ser372 is appropriate positioned to form a hydrogen bond with the pyridine nitrogen of Pon, and the deeper substrate binding pocket due to the presence of Ser372 may allow aromatic stacking of Trp220 with the pyridine ring of Pon in Pnao. Tyr218 and Thr381 in NicA2 form hydrogen bonds with the nitrogens of nicotine, whereas Asn224 and Phe338, respectively, are present at these positions in Pnao. Asn224 is oriented away from the substrate binding pocket in Pnao due to the presence of Trp240 (Leu234 at this position in NicA2), which occupies the space filled by Tyr218 in NicA2, and Asn224 may therefore not be able to interact with the substrate in Pnao. Overall, there are several differences in the active sites of NicA2 and Pnao, which presumably allow them to accommodate their different substrates.

### Structure of CycN and docking models

**Figure 12:**
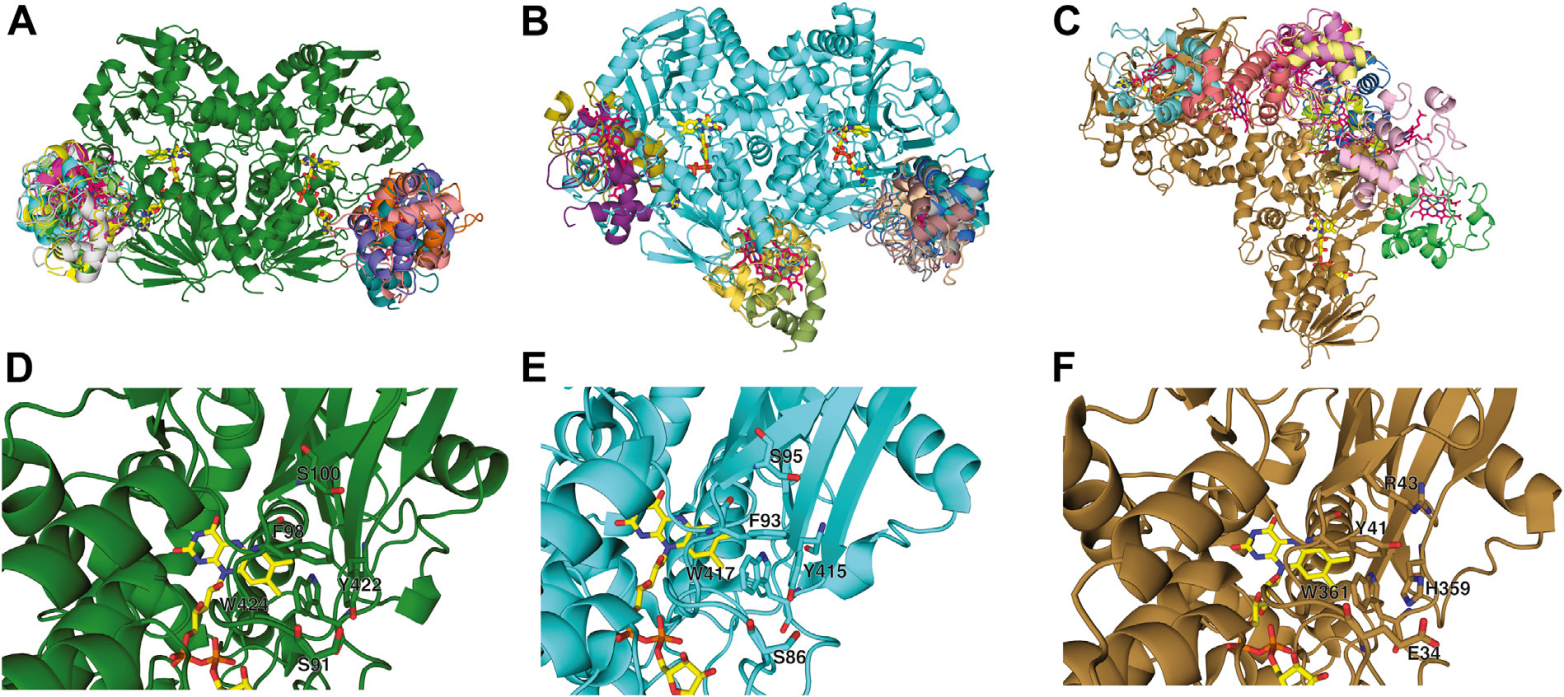
**Docking models.** (*A*), (*B*), and (*C*) ZDOCK docking models of CycN docked with Pnao, NicA2, and LHNO, respectively. Displayed are the top 10 docking poses for CycN with each enzyme. Pnao, NicA2, and LHNO are shown in *green*, *cyan*, and *sand*, respectively, and FAD is shown in *yellow* in all models. CycN in each docking pose is shown in a different color. Note that Pnao, NicA2 and LHNO are homodimers and there are therefore two equivalent potential CycN binding sites in each structure. The dimer interface of LHNO is drastically different from that of Pnao and NicA2. (*D*), (*E*), and (*F*) surface residues on Pnao, NicA2, and LHNO, respectively, at the region where CycN commonly docked with Pnao and NicA2. Residue numbering for all three enzymes is based on the full-length sequence, including signal peptides. FAD, flavin adenine dinucleotide; LHNO, L-6-hydroxynicotine oxidase; NicA2, nicotine oxidoreductase; Pnao, pseudooxynicotine amine oxidase.

We were able to solve the structure of CycN at 1.9 Å (Table S1), and the structure has a similar overall fold as bovine cytochrome *c*. We used rigid-body docking between CycN and Pnao and NicA2 and LHNO *via* the ZDOCK server to gain insight into how CycN may interact with these proteins (14). We used the structure of the homodimer for Pnao, NicA2, and LHNO as a basis for the docking models since these enzymes exist as homodimers in solution (6, 15, 16). The ZDOCK server returns the top 10 docking poses as an output, and for Pnao, all 10 docking poses had CycN positioned in the same spot on the surface of Pnao (Fig. 12*A*). Docking CycN with the structure of NicA2 produced similar results (Fig. 12*B*), with eight of the top 10 docking poses having CycN positioned at the same relative location as that seen in the Pnao docking poses. Interestingly, this was not observed when we performed docking of CycN with LHNO; in that case, CycN was distributed across several positions on the surface of LHNO, with only one of the top 10 docking poses showing CycN at the common region observed with NicA2 and Pnao (Fig. 12*C*). This observation is consistent with the fact that LHNO reacts poorly with CycN and suggests that this common docking position observed with NicA2 and Pnao may correspond to the true CycN binding site for these enzymes. The dimer interface of LHNO is drastically different from that of NicA2 and Pnao. However, this difference in dimer interface does not occlude in LHNO, the common CycN docking position observed in NicA2 and Pnao. Notably, several of the surface exposed residues in this putative CycN binding site are conserved between NicA2 and Pnao yet are different in LHNO, even though the sequence identity between these three proteins is in the range of 33 to 39%. For example, Pnao contains Ser91, Phe98, Ser100, and Tyr422 on the surface of this potential binding site, and NicA2 contains identical residues at these positions (Fig. 12, *D* and *E*). In contrast, LHNO has Glu34, Tyr41, Arg43, and His359 at the equivalent positions (Fig. 12*F*).

## Discussion

**Figure 13:**
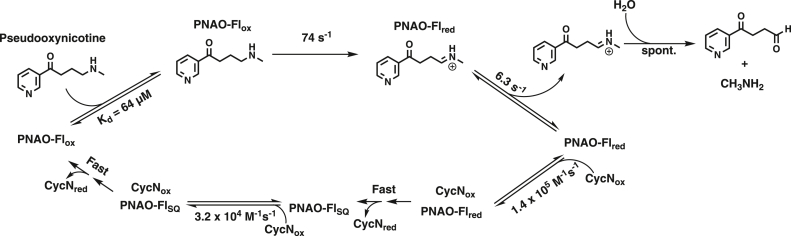
**Proposed kinetic model for Pnao’s catalytic cycle.** Pnao, pseudooxynicotine amine oxidase.

Our studies have investigated the structural and kinetic characteristics of Pnao and have allowed us to propose a kinetic model for Pnao’s catalytic cycle (Fig. 13). Two kinetic events are observed in the reductive half-reaction, with hydride transfer from Pon to Pnao’s flavin followed by dissociation of the imine-containing product. Comparing the k_cat_ from steady-state kinetics with our transient kinetic data suggests this product dissociation step primarily limits catalytic turnover by Pnao at saturating concentrations of CycN. Pnao-Fl_red_ at the end of the reductive half reaction must then be oxidized, and we evaluated both O_2_ and CycN as potential oxidants for the enzyme. While O_2_ can oxidize Pnao-Fl_red_, it does so quite slowly, with a rate constant of 600 M^-1^s^-1^, which is orders of magnitude lower than that of typical flavoprotein amine oxidases. In contrast, CycN oxidizes Pnao-Fl_red_ rapidly in under a second, with a rate constant that is comparable to that of *bona fide* oxidases with O_2_ (9). Pnao-Fl_red_ oxidation by CycN occurs in two kinetic events that each appear to be kinetically controlled by a bimolecular event. Since Pnao-Fl_red_ has two electrons to give and CycN is an obligate one electron acceptor, this is consistent with two stepwise one electron transfers from Pnao-Fl_red_ to two CycN molecules. Similar behavior was observed when NicA2-Fl_red_ is oxidized by CycN (6). The k_obs_ values for the two oxidation events by CycN both increased linearly with the CycN concentration, indicating that each electron transfer is rate-limited by binding of CycN_ox_ to Pnao-Fl_red_ and Pnao-Fl_SQ_ in the first and second events, respectively; as a result, the subsequent electron transfer between Pnao’s flavin and CycN’s heme after the complex has formed must be dramatically faster than binding. Notably, only the rate constant for the reaction between Pnao-Fl_red_ and CycN_ox_ should be considered when comparing CycN with O_2_ as an oxidant for Pnao because once Pnao-Fl_SQ_ has been formed, the rate of oxidation by O_2_ drops to near zero (Fig. 2). Thus, the rate constant for Pnao-Fl_red_ oxidation by CycN is 230-fold greater than the rate constant for oxidation by O_2_. In addition, we found that growth of *P. putida* S16 using Pon as a carbon source is severely limited in the absence of CycN. These findings strongly suggest that Pnao, like NicA2, is not an oxidase despite its name but rather a dehydrogenase that uses CycN as its oxidant both *in vivo* and *in vitro*.

The biological rationale for NicA2 and Pnao using CycN as an oxidant instead of molecular oxygen seems obvious, as it would allow *P. putida* S16 to funnel the electrons from nicotine and Pon oxidation into the electron transport chain, allowing additional energy production when the organism catabolizes nicotine. However, the mechanistic basis for this oxidant switch remains a mystery. Both NicA2 and Pnao have the same overall structural fold as countless other flavoprotein amine oxidases that react rapidly with O_2_ (12).The local environment surrounding the isoalloxazine of the flavin is also similar between Pnao, NicA2, and other known oxidases like LHNO, with Pnao and NicA2 even having the strictly conserved lysine residue near N5 of the isoalloxazine (K345 in Pnao and K340 in NicA2) that has been shown to be critical for the potent oxidase activity in other flavoprotein amine oxidases (11, 17). Pnao has aromatic cage residues that are typical of amine oxidases that are readily oxidized by O_2_ whereas NicA2 has an unusual Asn462 as one of the two aromatic cage residues. However, recent work demonstrated that this Asn residue is not responsible for limiting NicA2’s oxidase activity (18). The reaction between flavin hydroquinones and O_2_ in flavoprotein oxidases is thought to require an obligate flavin semiquinone-superoxide intermediate that can then undergo a second one-electron transfer to produce oxidized flavin and hydrogen peroxide (5, 9, 12). Both NicA2 and Pnao populate an obvious flavin semiquinone state during titrations with dithionite, indicating that both enzymes can stabilize the flavin intermediate needed to react with O_2_, yet both enzymes’ flavin hydroquinones are oxidized slowly by O_2_. Interestingly, LHNO does not produce a flavin semiquinone upon titration with dithionite, yet its flavin hydroquinone reacts rapidly with O_2_. Our previous work on NicA2 demonstrated that the N-methylmyosmine product stays tightly bound to NicA2 when the enzyme is oxidized by O_2_ or CycN, and then the product is rapidly released after flavin oxidation. Accordingly, product release was not rate limiting during turnover for NicA2. With Pnao, product release does appear to limit the rate of turnover.

The ability to use CycN as an oxidant is clearly not a general property of flavoprotein amine oxidase enzymes, as LHNO is poorly oxidized by CycN. We have identified a potential binding site for CycN on the surface of NicA2 and Pnao through protein–protein docking that has several strictly conserved residues between these two CycN-utilizing enzymes. However, the isoalloxazine is buried in both NicA2 and Pnao, with the C7–C8 edge of the isoalloxazine >10 Å away from the protein surface in both enzymes. This implies that conformational changes or long-range electron transfers are required for either of these enzymes to be oxidized by CycN. Notably, both NicA2 and Pnao have a Trp residue (Trp417 in NicA2 and Trp424 in Pnao) adjacent to the C7–C8 edge of the isoalloxazine that is next to a Tyr residue (Tyr415 in NicA2 and Tyr422 in Pnao) on the protein surface (Fig. 12*, D* and *E*). Trp and Tyr residues are capable of transferring single electrons between redox cofactors (19), and it is therefore tempting to speculate that these conserved residues in NicA2 and Pnao may comprise a protein-derived wire to transfer electrons from the reduced flavin to CycN on the surface of the enzymes. We intend to evaluate this potential role for these residues in future studies.

The flavin semiquinone of Pnao is remarkably resistant to reduction by dithionite. Given the low reduction potential of dithionite (*E*° = −660 mV) (20), this is most likely due to a kinetic block on reduction of Pnao’s semiquinone to the hydroquinone. A similar kinetic block on reduction to the hydroquinone has been observed before in *Methylophilus methylotrophus* electron transferring flavoprotein (ETF) (21). In that case, an arginine residue that sits over the flavin was shown to be responsible for the kinetic block, as mutating the arginine to alanine allowed the mutant ETF to be reduced to the hydroquinone by dithionite. In the structure of ETF, the isoalloxazine is surrounded by protein side chains, blocking access to solvent, which is likely a consequence of ETFs biological function as an electron mediator. In contrast, the *re* face of the isoalloxazine in Pnao is uncovered and exposed to the Pon binding site of Pnao (Fig. 11*A*). Our structure of Pnao contained oxidized FAD, and it therefore remains possible that a change in conformation occurs upon reduction to the semiquinone that occludes the isoalloxazine from the solvent and reduction by dithionite. Notably, NicA2’s flavin semiquinone can be reduced by dithionite to the hydroquinone (6). NicA2 stabilizes the neutral semiquinone, in contrast with the anionic semiquinone observed in Pnao. This may be due to the presence of the Asn462 side chain in the active site of NicA2, which is in hydrogen bonding distance to O4 of the isoalloxazine (Fig. 11*B*). It is unclear if this difference in semiquinone protonation state between the two enzymes has an impact on preventing the semiquinone from being reduced to the hydroquinone in Pnao. However, the low reactivity of the Pnao semiquinone appears to provide some added protection against reacting with O_2_ in favor of transferring electrons to CycN. The reduction potential for the Pnao-Fl_ox_/Pnao-Fl_SQ_ couple (-111 mV at pH 7.5) is higher than the O_2_/superoxide couple (-180 mV at pH 7) (22), indicating that thermodynamics can contribute to the very slow reactivity of Pnao-Fl_SQ_ with O_2_. Given how buried FAD is in the structure of Pnao, we suspect that CycN binds on the surface of Pnao, and the electrons on Pnao’s flavin are transferred to CycN over longer distances. Thus, electron transfer from Pnao to CycN would not be impeded by the kinetic block that prevents dithionite from reacting with the semiquinone in Pnao.

**Figure 14:**
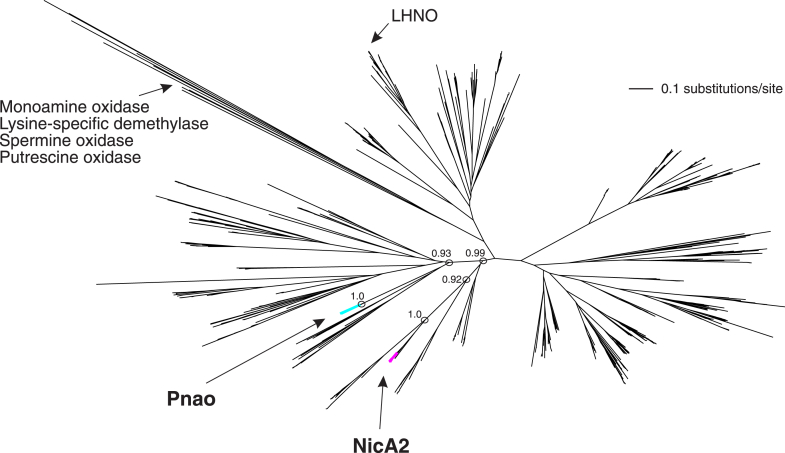
**A phylogenetic analysis of 869 bacterial flavin-dependent oxidases reveals that NicA2 and Pnao are independently evolved.** The NicA2 and Pnao branches are shown in *magenta* and *turquoise*, respectively. The well-studied monoamine oxidase from *Homo sapiens* and other enzymes are shown for reference. Most of the unlabeled branches represent predicted bacterial flavin-dependent enzymes that remain uncharacterized at the biochemical level. Branch support is shown for key nodes that separate Pnao from NicA2. NicA2, nicotine oxidoreductase; Pnao, pseudooxynicotine amine oxidase.

Given that NicA2 and Pnao are both from the same enzyme family and form an operon together with *cycN*, we fully expected that they would be recent duplicates and would appear as sister sequences in an evolutionary tree. To our surprise, a phylogenetic analysis of more than 800 diverse flavin-dependent amine oxidase homologs reveals that NicA2 and Pnao enzymes evolved as members of divergent lineages with each being more closely related to proteins from other bacterial species (Fig. 14). However, to our knowledge, none of the homologs between the NicA2 and Pnao lineages have yet been characterized for their substrate preference or oxidant specificity. Because both enzymes from *P. putida* S16 appear to be only distantly related to the *Arthrobacter* enzyme known to oxidize 6-L-hydroxynicotine and reduce dioxygen, it is still unclear if NicA2 and Pnao independently evolved their specificity for CycN or if they both evolved from a common, cytochrome c-utilizing ancestor. Ultimately, more work will be necessary to understand how these two enzymes can transfer electrons to a cytochrome c protein while most members of the enzyme family use O_2_ instead.

## Concluding remarks

The transient kinetic data in this study demonstrate that Pnao is oxidized by CycN 230 times faster than O_2_ at equivalent oxidant concentrations. This, combined with the fact that *cycN* is required for robust growth of *P. putida* S16 on Pon, strongly suggests that Pnao is a dehydrogenase that uses CycN as its physiological electron acceptor. We therefore propose renaming Pnao as Pon amine dehydrogenase. Importantly, our results indicate that, in the future, it should no longer be assumed that all enzymes with the flavoprotein amine oxidase structural fold use dioxygen to reoxidize their flavin.

## Experimental procedures

### Pnao expression and purification

The gene for Pnao without the signal peptide (from Ala45 onward) was synthesized by GenScript with codon optimization for *E. coli*. This gene was cloned into a pET28a vector containing an N-terminal His-SUMO tag, and this expression vector was transformed into *E. coli* BL21 (DE3) cells and grown in 4 L of protein expression media (12 g/L tryptone, 24 g/L yeast extract, 40 ml/L glycerol, 2.13 g/L K_2_HPO_4_, and 12.54 g/L KH_2_PO_4_) at 37 °C with shaking till an *A*_600_ of 0.8. The temperature was decreased to 20 °C, and cultures were induced with 100 μM IPTG and left to grow overnight. Pellets from harvested cultures were resuspended in 300 mM NaCl, 50 mM NaPO_4_ pH 8, 10% glycerol (lysis buffer) with 20 mM Imidazole. Cells were lysed by sonication after the addition of EDTA-free protease inhibitor cocktail (Abcam) and benzonase nuclease (Sigma). The lysate was cleared by centrifugation, and the supernatant was loaded on a nickel affinity column that had been pre-equilibrated with lysis buffer. Column was washed with ∼70 ml lysis buffer, and Pnao was eluted with lysis buffer + 250 mM imidazole. To the elution, ∼10 μM SUMO protease (ULP1) was added to cleave the His-SUMO tag. The elution was buffer exchanged to lysis buffer to remove imidazole, and the Pnao was run over the nickel column equilibrated with lysis buffer to remove the cleaved His-SUMO tag. Pnao was concentrated and then further purified using a HiLoad 16/600 Superdex 200 pg column in 40 mM Hepes, 100 mM NaCl pH 7.5, 10% glycerol (stopped-flow buffer). Purified protein was concentrated and flash frozen.

### Cytochrome c expression and purification

*E. coli* BL21 (DE3) cells were transformed with pEC86 helper vector and pET22b-*cycN* (6) and grown in 4 L of LB at 37 °C with shaking to an *A*_600_ of 0.6. The temperature was lowered to 30 °C and induced with 10 μM IPTG before being left overnight to grow. Cultures were harvested by centrifugation at 8000 rpm at 4° for 20 min. The supernatant was discarded, revealing pink pellets. Pellets were resuspended and lysed through osmotic shock using ice-cold osmotic shock buffer (0.5 M sucrose, 0.2 M Tris-HCl pH 8.0, and 0.5 mM EDTA), 50 ml buffer per liter of culture, then adding 33 ml of ice-cold water after resuspension. The mixture was left to gently agitate in a cold environment for 2.5 h. Resuspension mixture was spun down at 8000 rpm for 20 min at 4 °C, and the resulting pink supernatant was dialyzed overnight against 20 mM citric acid buffer pH 4. Dialyzed supernatant was spun down at 5000 rpm for 5 min at 4 °C to remove aggregated protein. The supernatant was loaded onto a SP Sepharose cation exchange column equilibrated in the same buffer and eluted using a linear NaCl gradient (0–1 M NaCl in 20 mM citrate pH 4). Potassium ferricyanide was then added to fully oxidize CycN. Excess ferricyanide was removed while running protein through a HiLoad Superdex 16/600 75 pg size-exclusion column equilibrated with stopped-flow buffer.

### Testing semiquinone stability

Pnao was made anaerobic in a cuvette by cycling between vacuum and argon, as previously described (20). Anaerobic dithionite was placed in a gas tight syringe and attached to the anaerobic cuvette. Dithionite was titrated into Pnao until the anionic semiquinone was reached, after which system was exposed to oxygen. Spectrophotometric scans were taken every 5 min for 2 hours to observe stability.

### Flavin reduction potential measurements

Spectrophotometric measurements of Pnao were taken using Shimadzu UV-1900 UV–Vis spectrophotometer at 25 °C. Reduction potentials were determined using the xanthine/xanthine oxidase method developed by V. Massey (8). In an anaerobic cuvette, a solution consisting of xanthine (0.3 mM), benzyl viologen (1 μM), and Pnao-Fl_ox_ (Abs_450_ = 0.4) in stopped-flow buffer was made anaerobic by cycling between vacuum and argon. Indigo disulfonate (Abs_600_ ∼ 0.4) was used as the indicator dye (E_m_=-139 mV at pH 7.5), and a catalytic amount of xanthine oxidase was added to initiate the experiment. Absorbance measurements were taken every 15 min for 24 h. Data showing absorbance changes of the enzyme and dye were fit to the Nernst equation (Equation 1), where F is Faraday’s constant, *n* is the number of electrons transferred to Pnao, R is the gas constants, and *T* is the temperature in Kelvin.

(1) $$\text{ln}{\left[\frac{\text{reduced}}{\text{oxidized}}\right]}_{\text{enz}}=\left({\text{E}}_{\text{m},\text{Dye}}-{\text{E}}_{\text{m},\text{Enz}}\right)\frac{n\text{F}}{\text{RT}}+\text{ln}{\left[\frac{\text{reduced}}{\text{oxidized}}\right]}_{\text{dye}}\;$$

### Transient kinetic assays

All stopped flow experiments were completed in stopped flow buffer at 4 °C using a TgK Scientific SF-61DX2 KinetAsyst stopped-flow instrument. All stopped-flow experiments were monitored using the instrument’s multiwavelength CCD detector. For the reductive half-reaction experiments, oxidized Pnao at ∼35 μM (before mixing) was placed in a tonometer and made anaerobic through cycling the solution between vacuum and argon, as previously described (20). The tonometer was loaded onto the instrument, and the enzyme was mixed against various concentrations of Pon (0.05–1 mM before mixing) in buffer that had been sparged with argon to achieve anaerobiosis. For the oxidative half-reaction experiments, reduced Pnao was prepared by titrating ∼35 μM Pnao with anaerobic Pon in a gas tight syringe until the hydroquinone was reached based on UV/Vis spectrum, after which the tonometer was loaded on the instrument. For experiments monitoring the reoxidation by O_2_, different O_2_ concentrations were prepared by bubbling buffer with various O_2_/N_2_ ratios (prepared with a gas blender) in a gas tight syringe. For experiments monitoring the reoxidation by CycN, CycN solutions were made anaerobic in a tonometer by cycling with vacuum and argon before loading on the instrument and mixing with Pnao-Fl_red_. CycN concentrations were determined using the extinction coefficient of oxidized cytochrome *c* at 410 nm (101, 600 M^-1^ cm^-1^). A similar assay was performed using bovine cytochrome *c* (Sigma-Aldrich). Initial absorbance reading of ∼10 μM of bovine cytochrome *c* was taken using a Shimadzu UV-1900 UV–vis spectrophotometer. Changes in absorbance were observed after the stepwise addition of 100 μM Pon and 100 nM Pnao. The same experiment was repeated with yeast cytochrome *c* (Sigma Aldrich).

Stopped-flow data were analyzed using KaleidaGraph. Traces for the reaction with O_2_ were fit to a single exponential function (Equation 2) to determine observed rate constant (k_obs_) values for each O_2_ concentration. Traces for the reaction with Pon were fit to a sum of two exponentials (Equation 3) to determine k_obs_ values for the first and second phase, whereas traces for the reaction with CycN were fit to a sum of three exponentials (Equation 4). In Equation 2, Equation 3, and Equation 4, ΔA is the kinetic amplitude for each phase, k_obs_ is the apparent first order rate constant, and A_∞_ is the absorbance at the end of the reaction.

(2) $$Y=\Delta Ae^{-k_{obs}t}+A_{\infty }\;$$

(3) $$Y=\Delta A_{1}e^{-k_{obs1}t}+\Delta A_{2}e^{-k_{obs2}t}+A_{\infty }$$

(4) $$Y=\Delta A_{1}e^{-k_{obs1}t}+\Delta A_{2}e^{-k_{obs2}t}+\Delta A_{3}e^{-k_{obs3}t}+A_{\infty }$$

Plots of k_obs_ against substrate concentration were fit to a line for the reactions with O_2_ and CycN. The plot of k_obs1_ against [Pon] displayed a hyperbolic dependence and was fit to Equation 5 to determine k_red_, the rate constant for flavin reduction, and K_d_ for Pon binding to Pnao-Fl_ox_.

(5) $$k_{obs}=\frac{k_{red}\left[S\right]}{K_{d}\;+\;\left[S\right]}$$

### Steady-state kinetic assays

All experiments were completed in stopped flow buffer at 4 °C using a TgK Scientific SF-61DX2 KinetAsyst stopped-flow instrument. Pnao was placed into a tonometer with a Pon solution in a side arm, and the tonometer made anaerobic through cycling between argon and vacuum. Once anaerobic, Pnao and Pon were mixed to achieve a Pnao concentration of 200 nM and 2 mM Pon in the tonometer before mixing with CycN. The solution was then mixed with various concentrations of anaerobic, oxidized CycN (5 μM to 160 μM before mixing). The reduction of CycN was monitored at 550 nm to determine initial velocities. Data were collected using Kinetic Studio Software and evaluated using KaleidaGraph. 21,000 M^-1^ cm^-1^ was used as the difference in extinction coefficient at 550 nm between oxidized and reduced CycN (6), and the rate was divided by two to take into consideration that two molecules of cytochrome c are involved during a single turnover by Pnao.

### Plate reader growth assay

Bacteria containing pJN105 plasmid were maintained under 25 μg mL^-1^ gentamicin selection where noted. *P. putida* S16 was electroporated with either pJN105 empty vector or pJN105-*cycN* and plated onto LB-gentamicin to grow at 30 °C overnight. Five distinct colonies were picked as biological replicates and grown in LB-medium supplemented with gentamicin shaking at 30 °C overnight. The next day, overnight cultures were subcultured into minimal media (6 g L^-1^ sodium phosphate dibasic, 3 g L^-1^ potassium phosphate monobasic, 0.5 g L^-1^ sodium chloride, 1 g L^-1^ ammonium chloride, 0.4% glycerol, 1 mg L^-1^ thiamine, 1 mM MgSO_4_, 0.1 mM CaCl_2_, 1x trace metals mixture (Teknova), 25 μg mL^-1^ gentamicin) and grown into log phase. The *A*_600_ of cultures was determined, and all strains were adjusted to an *A*_600_ of 1.0 in minimal media without a carbon source. 2 μl of *A*_600_ 1.0 culture was added to 200 μl Pon media (Made with the same ingredients as minimal media, but with 0.5 g L^-1^ Pon as a carbon source instead of glycerol) supplemented with 0.2% arabinose or without arabinose in the case of the uninduced control, in a 96-well plate covered with a Breathe-easy sealing membrane (Sigma). The plate was set to shake at 30 °C, and absorbance monitored at 600 nm in a Tecan M200 plate reader for 2 days.

### Pnao crystal studies

Preliminary screening for crystallization was completed using the National High-Throughput Crystallization Center at the Hauptman-Woodward Institute (23). After optimization screening, crystals for data collection were grown at room temperature using a hanging drop vapor diffusion method by mixing protein (1uL of 10 mg/ml) and an equal amount of reservoir well solution (0.1 M KBr + 0.1 M TAPS pH 9 + PEG 4000 40%). Crystals were harvested and cryoprotected by replacing the water content with ethylene glycol. Crystal diffraction data were collected at the Life Sciences Collaborative Access Team beamline 21-ID-G at the Advanced Photon Source, Argonne National Laboratory. Data integration and scaling were performed with iMosflm (24) and AIMLESS (25), respectively. The space group was determined to be P2_1_ with unit cell parameters a = 143.99 Å, b = 50.34 Å, c = 150.89 Å, which suggests an asymmetric unit containing four molecules. The Pnao structure was solved by molecular replacement using PHENIX Phaser-MR (26) with NicA2 structure (PDB: 6C71) as a search model (27). Multiple rounds of structural refinement and manual model building were performed in PHENIX Refine program (28) and Coot (29). Crystallographic data and refinement statistics are given in Table S1.

### CycN crystal studies

CycN was dialyzed into deionized H_2_O and supplemented with potassium ferricyanide to ensure total oxidation of the protein. The end solution had 1.3 mM (15 mg/ml) CycN and 5 mM potassium ferricyanide. This solution was mixed 1:1 with 0.1 M MES monohydrate pH 6.0, 20% polyethylene glycol monomethyl ether 2000, and set to incubate at 20 °C using the hanging drop vapor diffusion method until crystal formation. Crystals were harvested and flash frozen in the same buffer with an added 20% glycerol as cryoprotectant. Crystal diffraction data were collected at the Life Sciences Collaborative Access Team beamline 21-ID-F at the Advanced Photon Source, Argonne National Laboratory. Data integration and scaling were performed as described earlier for Pnao. The space group was determined to be C2 with unit cell parameters a = 79.73 Å, b = 30.17 Å, c = 49.10 Å, which suggests an asymmetric unit containing a single molecule. The CycN structure was solved by molecular replacement using PHENIX Phaser-MR (26) with a horse cytochrome c structure (PDB: 1CRC) as a search model (30). Multiple rounds of structural refinement and manual model building were performed as described for Pnao. Crystallographic data and refinement statistics are given in Table S1.

### Phylogenetic analyses

In order to determine the relatedness of Pnao and NicA2 relative to one another and to other flavin-dependent protein sequences, we used BLAST analyses of the non-redundant database of GenBank. Alignment of the 869 amino acid sequences obtained was achieved using MAFFT version 7 (31) using the auto search strategy which maximizes both accuracy and speed. A maximum likelihood phylogenetic estimate for the flavin-dependent family members was obtained using FastTree (32) assuming the JTT model for amino acid substitution with a CAT approximation with 20 rate categories to accommodate among-site rate heterogeneity. Local branch support was estimated using the Shimodaira-Hasegawa test (33) using 1000 resamples as implemented in FastTree.

## Data availability

The coordinates for the structure of Pnao and CycN have been deposited in the Protein Data Bank under PDB ID 7U6L and 7TLX.

## Supporting information

This article contains supporting information

## Conflicts of interest

The authors declare that they have no conflicts of interest with the contents of this article.
