# Supplementary material for: The enzyme pseudooxynicotine amine oxidase from Pseudomonas putida S16 is not an oxidase, but a dehydrogenase
Source: J Biol Chem. 2022 Jul 11;298(8):102251. doi: 10.1016/j.jbc.2022.102251 (PMC9396064; doi:10.1016/j.jbc.2022.102251)
Supplement: Table S1 [file mmc1.docx]

**Table S1.** Data collection and refinement statistics.

|  | Pnao (PDB ID: 7U6L) | CycN (PDB ID: 7TLX) |
| --- | --- | --- |
| **Data Collection** | |  |
| Wavelength (Å) | 0.98 | |
| Resolution range | 47.86 - 2.6 | 41.84-1.80 |
| Space group | P 1 2_1_ 1 | C2 |
| Unit cell a, b, c (Å) a, b, g (°) | 143.99, 50.34, 150.89  90.0, 107.9, 90.0 | 79.73, 30.17, 49.10  90.0, 121.6, 90.0 |
| Total reflections | 242390 | 107297 |
| Unique reflections | 64421 | 9383 |
| Multiplicity | 3.8 | 3.6 |
| Completeness (%) | 100 (98.4) | 99.9 (99.7) |
| Mean I/sigma(I) | 8.3 | 8.2 |
|  |  |  |
| R_merge_ (%) | 12.3 (42.8) | 5.6 (17.2) |
| R_meas_ (%) | 14.3 (49.8) | 7.8 (23.8) |
| CC1/2 | 0.989 (0.839) | 0.996 (0.963) |
| **Refinement** | |  |
| Reflections used in refinement | 64421 (6149) | 9379 (927) |
| R-work (%) | 18.3 (23.9) | 17.4 (19.0) |
| R-free (%) | 23.4 (30.1) | 19.1 (20.9) |
| Number of non-hydrogen atoms | 13786 | 856 |
| macromolecules | 13457 | 740 |
| ligands | 336 | 43 |
| solvent | 117 | 73 |
| Protein residues | 1737 | 101 |
| RMS(bonds) | 0.007 | 0.013 |
| RMS(angles) | 0.92 | 1.09 |
| Ramachandran favored (%) | 94.77 | 97.98 |
| Ramachandran allowed (%) | 5.12 | 2.02 |
| Ramachandran outliers (%) | 0.12 | 0.00 |
| Rotamer outliers (%) | 2.28 | 0.00 |
| Clashscore | 13.21 | 4.00 |
| Average B-factor | 31.44 | 20.77 |
| Wilson B-factor | 27.87 | 15.12 |

Statistics for the highest-resolution shell are shown in parentheses.
